# The Band-Gap Studies of Short-Period CdO/MgO Superlattices

**DOI:** 10.1186/s11671-021-03517-y

**Published:** 2021-04-09

**Authors:** Ewa Przeździecka, P. Strąk, A. Wierzbicka, A. Adhikari, A. Lysak, P. Sybilski, J. M. Sajkowski, A. Seweryn, A. Kozanecki

**Affiliations:** 1 Institute of Physics, Polish Academy of Sciences, Al. Lotników 32/46, 02-668 Warsaw, Poland; 2 Institute of High Pressure Physics, Polish Academy of Sciences, Sokołowska 29/37, 01-142 Warsaw, Poland

**Keywords:** Superlattice, MgO, CdO, Energy bands manipulation, Molecular beam epitaxy, Crystal growth

## Abstract

Trends in the behavior of band gaps in short-period superlattices (SLs) composed of CdO and MgO layers were analyzed experimentally and theoretically for several thicknesses of CdO sublayers. The optical properties of the SLs were investigated by means of transmittance measurements at room temperature in the wavelength range 200–700 nm. The direct band gap of {CdO/MgO} SLs were tuned from 2.6 to 6 eV by varying the thickness of CdO from 1 to 12 monolayers while maintaining the same MgO layer thickness of 4 monolayers. Obtained values of direct and indirect band gaps are higher than those theoretically calculated by an ab initio method, but follow the same trend. X-ray measurements confirmed the presence of a rock salt structure in the SLs. Two oriented structures (111 and 100) grown on *c*- and *r*-oriented sapphire substrates were obtained. The measured lattice parameters increase with CdO layer thickness, and the experimental data are in agreement with the calculated results. This new kind of SL structure may be suitable for use in visible, UV and deep UV optoelectronics, especially because the energy gap can be precisely controlled over a wide range by modulating the sublayer thickness in the superlattices.

## Introduction

Wide band gap semiconductors like oxides and nitrides represent a family of semiconductors of crucial importance for modern optoelectronics, being used in short-wavelength light emitting diodes, laser diodes and optical detectors, as well as high-power, high-temperature, and high-frequency electronic devices such as field-effect transistors [1]. The energy band gap is a key factor in many fields of science, such as photovoltaics and optoelectronics. Ternary alloys can be obtained as random crystals or quasi-crystals short-period superlattices [2–5]. In the case of random crystals, in some systems there is a significant problem with obtaining materials in the full composition range without phase and concentration separation. This kind of problem has been reported in the case of ZnMgO and ZnCdO [6] oxide systems, especially because ZnO usually crystallizes in a wurtzite structure, whereas both CdO and MgO crystallize in a rock salt cubic structure [7]. Therefore, obtaining homogeneous alloys without crystal phase segregation in the middle composition range has proved to be a challenge in the case of these materials. This does not concern only oxides; a similar problem has also been reported, for example, in the case of InGaN [8].

CdO with a rock salt crystal structure is one of the transparent conductive oxides (TCOs). One of the major disadvantages of CdO is its relatively small intrinsic direct band gap of only 2.2 eV. Even though the Burstein–Moss effect caused by free carriers in the conduction band can shift the absorption edge to about 3 eV in most heavily doped CdO [9, 10], this is still not sufficient for the photovoltaic applications that utilize the UV part of the solar spectrum. Thus, opening the band gap of CdO will improve prospects for solar cell technologies. The cutoff working wavelength of solar-blind UV detectors should be shorter than 280 nm, corresponding to a band gap value of 4.5 eV [11], which is much larger than, for example, the band gap of pure CdO and ZnO (3.37 eV). Therefore, opening of the CdO band gap is also crucial for this field.

The use of superlattices can allow much more precise control of the composition, and good-quality ternary alloys in a wide range of compositions can be obtained in many semiconductor systems [2, 5, 12, 13]. Band gap engineering, crucial for the design of optoelectronic devices, can be realized in SLs by varying the layer thicknesses [3, 14]. A direct band gap of 2.5 eV has been reported for CdO, whereas in the case of MgO an energy gap of 7.8 eV was observed in a rock salt structure [15]. Theoretically, the rock salt cubic structure is stable over all (Mg,Cd)O compositions, as expected from the preferences of the binary oxides [16]. Usually, however, CdO layers are grown at much lower temperatures than MgO; thus it is a problem to obtain homogeneous mixed crystals over the full composition range. For this reason the number of reports on CdMgO alloys is very limited, and increasing the quantity of Cd can result in the presence of two compositions, as has been described in the case of CdMgO grown by metal organic chemical vapor deposition (MOCVD) [17]. CdMgO alloy thin films with total Mg concentration as high as 44% were obtained by magnetron sputtering [18]. In the case of layers obtained by the pulsed laser deposition technique the energy band gap of CdMgO was shifted to 3.4 eV [19], whereas in polycrystalline In-doped CdMgO films the maximum value of the energy gap was reported to be about 5 eV [20]. At the opposite end of the composition range, undoped and 1%, 2% and 3% Cd-doped MgO nanostructures were grown by the successive ionic layer adsorption and reaction (SILAR) method [21]. In the whole composition range only nanoparticles were obtained, but still in a range of Mg content of 0.34 ≤ *x* ≤ 0.84 the co-existence of two phases of Cd-rich and Mg-rich Cd_1−*x*_Mg_*x*_O is reported [22].

Most recent theoretical works are based on density functional theory calculations and are devoted mainly to the properties of binary compounds of CdO and MgO, including investigation of structural [23–25], electronic [26], spectroscopic [27], optical [28–30], magnetic [31–35] or other properties of doped compounds [36–38], Gorczyca et al. [13, 14] have conducted band gap engineering investigations of ZnO/MgO SL. No theoretical investigation of CdO/MgO superlattices has been reported in the literature, and this fact motivated us to study them.

In our previous work we have demonstrated the possibility of obtaining {CdO/MgO} SLs by Molecular Beam Epitaxy (MBE) [39]. In this study, we explore experimentally and theoretically methods for modulating the transparency of CdO-based TCOs by alloying this material with MgO, a larger band gap metal oxide with the same (rock salt) crystal structure. We grew {CdO/MgO} superlattice (SL) quasi-alloys by MBE in the whole composition range, and showed that the energy gap can be increased from 2.2 to 6 eV by changing the CdO sublattice thickness in these superlattices.

## Methods

Short-period {CdO/MgO} SLs were grown by plasma-assisted MBE (Compact 21 Riber) on differently oriented sapphire substrates: on *c*- and *r*-Al_2_O_3_. Before growth, the Al_2_O_3_ substrates were chemically cleaned and degassed in a buffer chamber at 700 °C. The substrates were then transferred to a growth chamber and annealed at 700 °C in oxygen (flow rate 3 ml/min). All of the multilayer structures were grown at 360 °C. Thin layers of CdO and MgO were deposited sequentially, and their thicknesses were estimated on the basis of growth conditions (numbers of periods in the individual samples were calculated to obtain the same final thickness of the samples). In the presented series of samples the thickness of the MgO sublayers is fixed, and we vary the thickness of CdO layers from ~ 1 to ~ 12 monolayers (ML).

A Panalytical X’Pert Pro MRD diffractometer was used to perform X-ray diffraction (XRD) analysis of the samples. The apparatus is equipped with a hybrid two-bounce Ge (220) monochromator, a triple-bounce Ge (220) analyzer, and two detectors: proportional and Pixcel. Two types of measurements were performed: *θ*/2*θ* scans at low-resolution settings in a wide angle range, and rocking curves, 2/*ω* scans and XRD reciprocal space maps at high-resolution settings.

Optical transmittance spectra were obtained at room temperature using a Varian Cary 5000 spectrophotometer, in a range from 200 to 700 nm. A two-channel measurement technique was used for transmittance measurements of the studied film. SL samples were placed in the measuring channel of the spectrophotometer, and the substrate (*r*- or *c*-oriented sapphire) was placed in the comparison channel.

## Results and Discussion

### Experimental Study

**Fig. 1:**
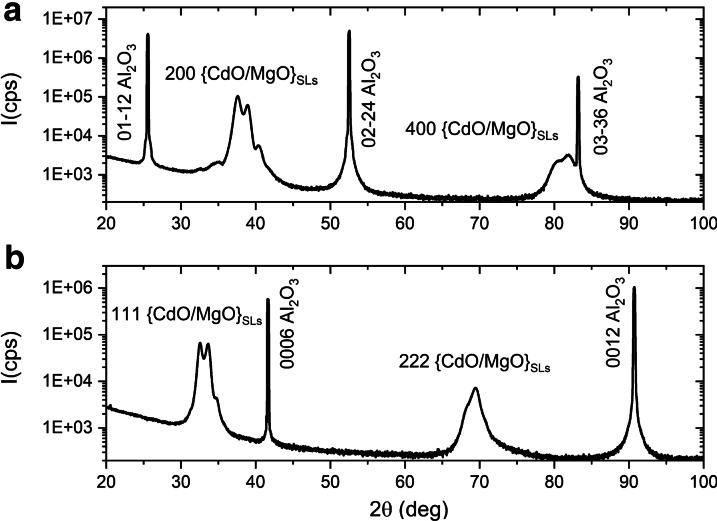
Theta–2Theta XRD scans of the {CdO(12.5 ML)/MgO(4 ML)} superlattices on **a** *r*-Al_2_O_3_ and **b** *c*-Al_2_O_3_

Superlattice structures with 4 ML MgO and with CdO sublattice thickness ranging from 1 to 12 ML were analyzed. Figure 1a, b show the full-range XRD scans for selected {CdO/MgO} SLs. The *θ*/2*θ* patterns indicated two crystallographic orientations of the substrate: [01-12] and [0001] (*r*-orientation and *c*-orientation). We also recorded a cubic phase of the {CdO/MgO} superlattices SLs. For the samples grown on *r*-plane sapphire substrate we obtained [100] {CdO/MgO} SLs orientation and for the structures grown on *c*-plane sapphire substrate we received [111] {CdO/MgO} SLs orientation. We do not observe other crystallographic phases of {CdO/MgO} materials.

**Fig. 2:**
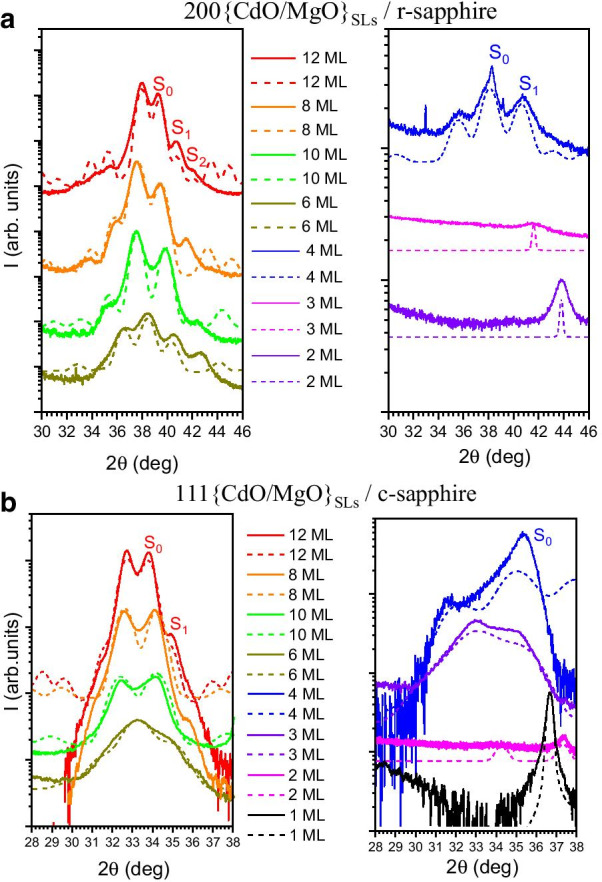
2Theta–Omega of 200 {CdO/MgO} on r-Al_2_O_3_ (**a**) and 111 {CdO/MgO} on c-Al_2_O_3_ (**b**) XRD peaks of the series of SLs with different CdO layers thickness. Solid lines are the 2*θ*/*ω* XRD scan measurement results and dash lines are 2*θ*/*ω* XRD scan simulations. On the legend we mark the amount of CdO monolayers (ML)

For thorough analysis of {CdO/MgO} SLs the 2 Theta–Omega (2*θ*/*ω*) scans in high resolution mode were measured. For the SLs structures grown on *r*-sapphire we investigated 200{CdO/MgO} X-ray diffraction reflection (Fig. 2a) and for the SLs structures grown on *c*-sapphire we investigated 111 {CdO/MgO} X-ray diffraction reflection (Fig. 2b). The solid lines on Fig. 2 shows the measurement results. Superlattice-related satellite peaks are clearly observed in both orientations, confirming the good periodicity and smoothness of the interfaces. Zero order peaks describing average parameters of SLs are marked as *S*_0_. Position of *S*_0_ peak depends on CdO sublayers thickness. Satellite peaks (*S*_1_, *S*_2_) are well defined in both samples. 2*θ*/*ω* XRD scans show that the main peak coming from SL (*S*_0_ order peak) is shifted to smaller angles with increasing of Cd concentration. It indicates that lattice parameters is increasing with higher Cd content.

For each measured 2*θ*/*ω* scan we calculate the 2*θ*/*ω* profiles using fitting procedure described in [40]. On Fig. 2 we show 2*θ*/*ω* XRD scan simulations by dashed lines. The simulation procedure is based on the dynamical theory of X-ray diffraction described by Takagi and Taupin [41–43]. We use X’Pert Epitaxy software provided by Malvern Panalytical company to simulate our 2*θ*/*ω* curves. The results obtained from simulated data we collected in Table 1.

**Table 1:** Thicknesses of individual layers in {CdO/MgO} superlattices on *r*-sapphire and on *c*-sapphire planed in MBE growth process and the best fit of XRD calculations

Samples on *r*-plane sapphire			Samples on *c*-plane sapphire		
MgO/CdO ML MBE	MgO/CdO thickness XRD (nm)	MgO/CdO ML XRD	MgO/CdO ML MBE	MgO/CdO thickness XRD (nm)	MgO/CdO ML XRD
4/12	2/3.25	4.8/11	4/12	1.8/7.1	4.3/15
4/10	2/2.2	4.8/4.7	4/10	1.73/3.6	4.1/7.7
4/8	2.1/2.9	4.9/6.3	4/8	1.81/4.1	4.3/8.7
4/6	2.6/2.4	6.1/5.1	4/6	2.25/3.17	5.3/6.7
4/4	2.15/1.6	5.1/3.4	4/4	2.25/2.2	5.3/4.7
4/3	2.5/1.1	5.9/2.5	4/3	2/1.2	4.8/2.5
4/2	2.15/1	5.1/1.5	4/2	2.25/0.8	5.3/1.7
			4/1	2.25/0.5	5.3/1.1

The most important parameter as we received from XRD simulations is the thickness of individual MgO and CdO layer in SL structure (Table 1). It is clearly visible that the thickness of MgO layer is equal to 2 nm for each sample as it was assumed during MBE growth process. For the CdO layers thickness we observe some differences with assumed parameters. The data present in Table 1 shows the recalculated thickness of individual CdO and MgO layers in SLs (from XRD simulations) expressed by amount of MLs.

**Fig. 3:**
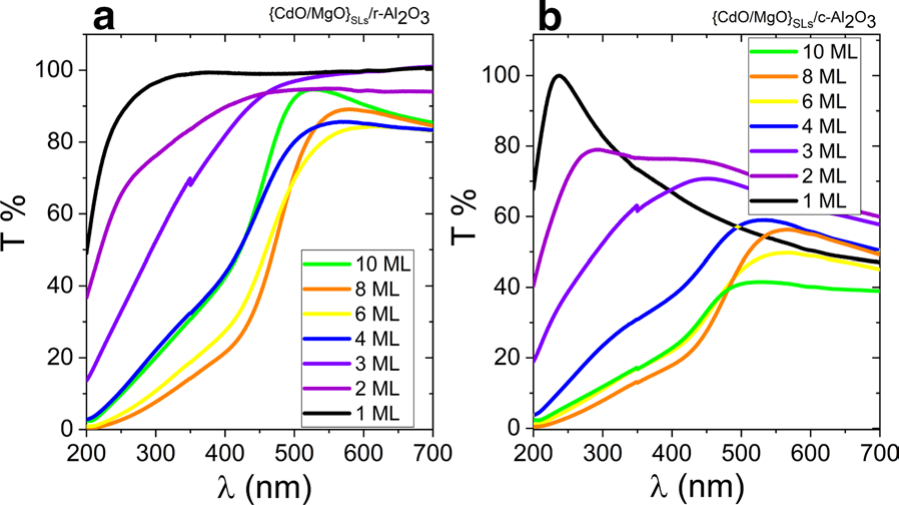
Transmittance of {CdO/MgO} SLs films on (**a**) *r*-sapphire and (**b**) *c*-sapphire

**Fig. 4:**
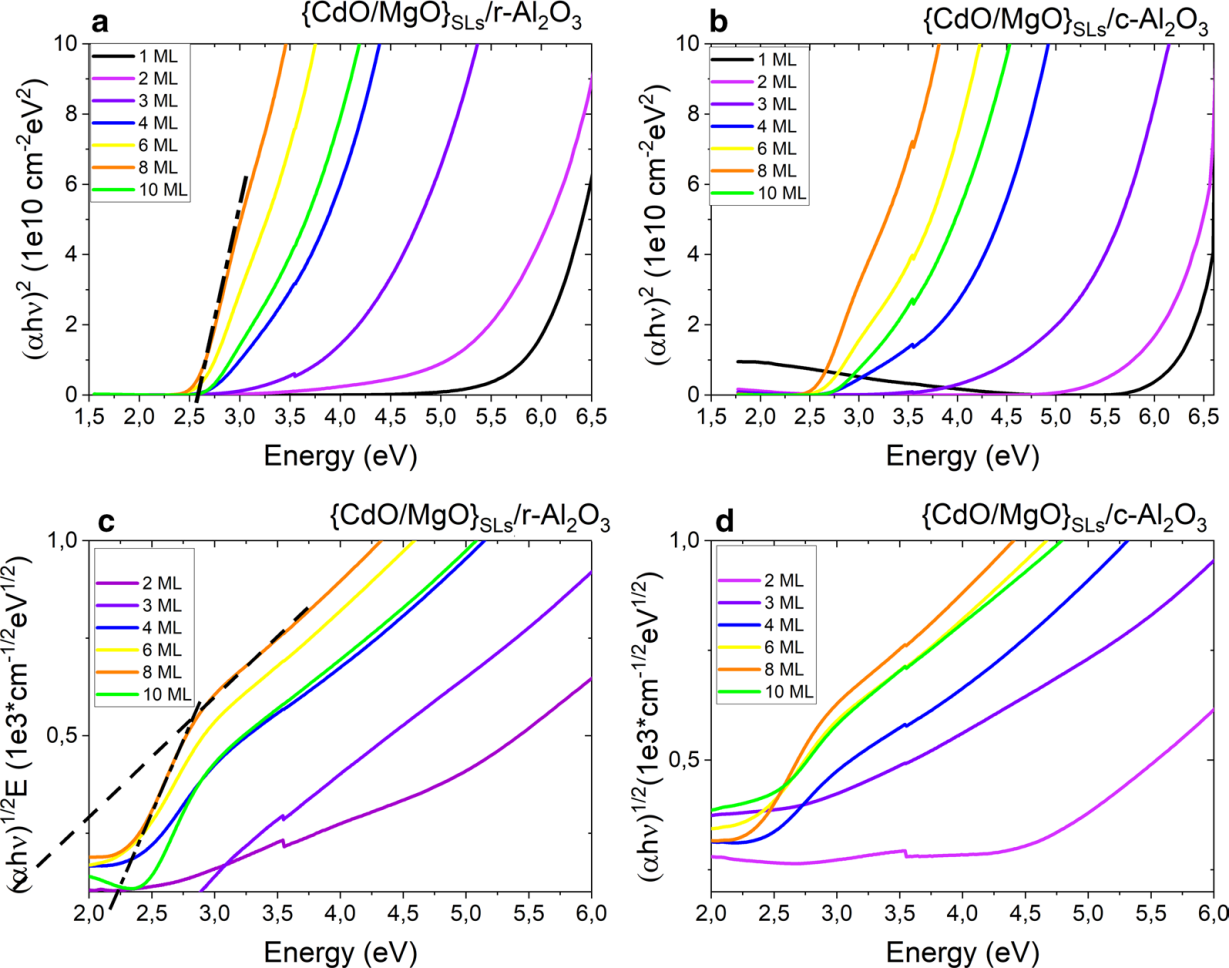
(*α* *hν*)^2^ and (*αhν*)^1/2^ plots as a function of photon energy (*hν*) for the {CdO/MgO} SLs films on *c*- or *r*-sapphire

The {CdO/MgO} quasi-alloy films were analyzed with a UV–visible–infrared spectrometer to study their energy band gaps. Figure 3 shows transmittance spectra measured at room temperature. The cutoff for transmission is continuously shifted to shorter wavelengths as the CdO sublayer thickness decreases. The transmittance drops in the NIR region may be related to free carrier absorption and plasma reflection [44]. As we know, CdO is highly conductive, in contrast to MgO. When the relative thickness of CdO with respect to MgO increases, most probably the resistivity of the samples increases due to the greater thickness of the CdO sublayers. Interestingly, the transmittance drop depends on the orientation of the SLs, which requires further research. The energy band gap values (*E*_g_) of SLs are derived by extrapolating the graph of *α*^2^ versus *hν* in the case of direct transitions (Fig. 4a, b) and that of *α*^1/2^ versus *hν* in the case of indirect transitions, where *α* is the absorption coefficient and *ν* is the photon frequency, according to the work of Tauc [45]. In samples with a higher CdO thickness, and thus with a relatively higher concentration of Cd in the CdMgO alloy, we can extract two indirect band gaps, with two linear regions as shown in Fig. 4c, d. Figure 4 shows that the band gaps of CdMgO decrease together with CdO thickness. The optical transmission measurements demonstrate that the direct energy band gap of {CdO/MgO} quasi-alloys can be varied over a range from 2.6 to 6 eV.

### Calculation Method

The Vienna ab Initio Simulation Package (VASP), based on quantum density functional formalism, following earlier investigations, was used in all calculations reported here [46–48]. Optimization of the ionic positions was performed in two stages, using different generalized gradient approximation (GGA) functionals for exchange–correlation energy. A standard plane wave functional basis set, with an energy cutoff of 605 eV, was used. The Monkhorst–Pack grid (5 × 5 × 5) was used for efficient integration in k-space [49]. Projector-Augmented Wave (PAW) pseudopotentials with Perdew, Burke, and Ernzerhof (PBE) exchange–correlation functionals were used in the treatment of Cd, Mg, and O atoms [50–52]. An electronic self-consistent (SCF) loop was terminated for a relative energy change below 10^–7^. The ab initio lattice parameters for bulk oxides were as follows: *a*_CdO_ = 4.783 Å, *a*_MgO_ = 4.236 Å. These lattice parameters are in good agreement with the values determined by X-ray measurements: *a*_CdO_ = 4.695 Å, *a*_MgO_ = 4.21 Å [15, 53]. The positions of the atoms were relaxed until the magnitude of the force acting on a single atom was below 0.005 eV/Å.

**Fig. 5:**
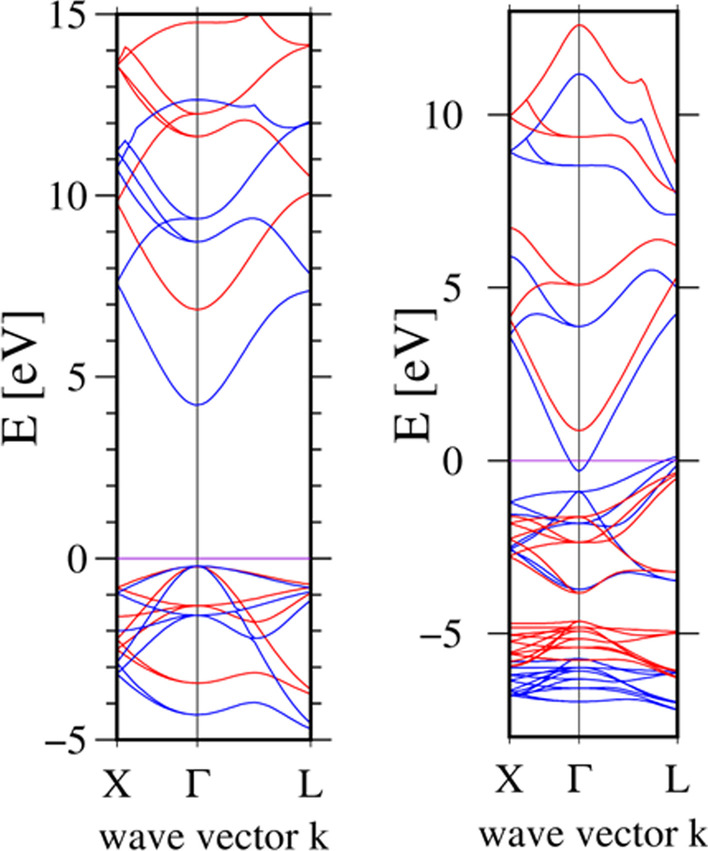
(Color online) Band structure obtained from VAPS for PBE (blue) exchange–correlation functional and GGA-1/2 (red) correction for MgO (left) and CdO (right)

The PBE density functional provides incorrect values for band gaps of semiconductors. Several methods have been used to remove this deficiency, such as the (GW) approximation [54], hybrid functionals using Hartree–Fock correction [55], or half-occupation generalized-gradient approximation (GGA-1/2) [56]. In the reported calculation we used the most efficient latter scheme, proposed by Ferreira et al. [56]. Spin–orbit effects were neglected in these calculations, since the high-lying valence states and low-lying conduction states lead to a small splitting (of the order of 10 meV). The calculated band gaps of bulk MgO and CdO were *E*_Γ_(MgO) = 7.1 eV and *E*_Γ,L_(CdO) = 2.55, 1.23 eV, respectively. Thus, satisfactory agreement with low-temperature experimental band gaps was obtained: *E*_g_(MgO) = 7.83 eV [15] and *E*_Γ, L_(CdO) =  ~ 2.5, 0.8–1.12 eV [57, 58]. This completes the above-mentioned second stage in which the final results are obtained by application of the modified GGA-1/2 correction method to structures in which the positions of atoms and a periodic cell size were determined in the first stage using the PBE approximation. The band structures of bulk MgO and CdO for PBE and GGA-1/2 approximations are shown in Fig. 5. It is seen that PBE underestimates the value of the energy gap, while in GGA-1/2 it is calculated correctly. After correction, the Fermi energy lay between the valence band maximum (VBM) and conduction band minimum (CBM). The band gap of CdO is consistent with the experimental measurements of Refs. [58] and [57], while the energy gap of MgO is consistent with Ref. [15]. The location of the Fermi level in CdO is the same as in a theoretical model based on the GW approach [59].

**Fig. 6:**
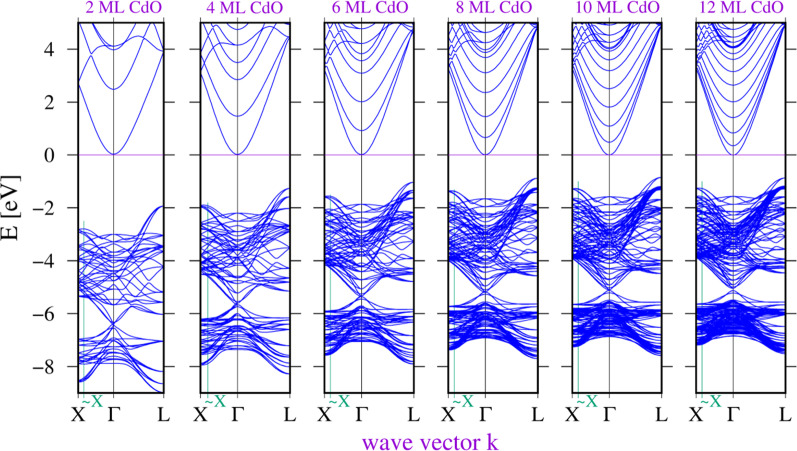
Calculated band structures of cubic {CdO/MgO} SLs for various numbers of CdO monolayers and for four monolayers of MgO, using the GGA-1/2 method

In the theoretical analysis of coherent CdO/MgO multiquantum wells, we used structures grown on the [001] direction. Layers of CdO and MgO were fully strained, i.e. there were single common lattice constants for the whole structure, and we assumed that there were no dislocations or defects at the interfaces between the two materials. The structure was relaxed using a conjugant gradient (CG) algorithm for force minimization. The Fermi energy was common for the whole structure, and as it was close to the CBM, the carrier concentration was set to 10^20^ cm^3^. We calculated common lattice constants for structures composed of 4 ML of MgO and CdO layers ranging from 2 to 12 ML. For these structures, we calculated energy gaps between different points in the Brillouin zone using the GGA-1/2 correction method. Figure 6 shows differences between the minimum of the conduction band and maxima in the valence band at the *X*, *L* points, and one maximum located close to the *X* point but shifted slightly towards the *X* point, which we have marked ~ *X*.

**Fig. 7:**
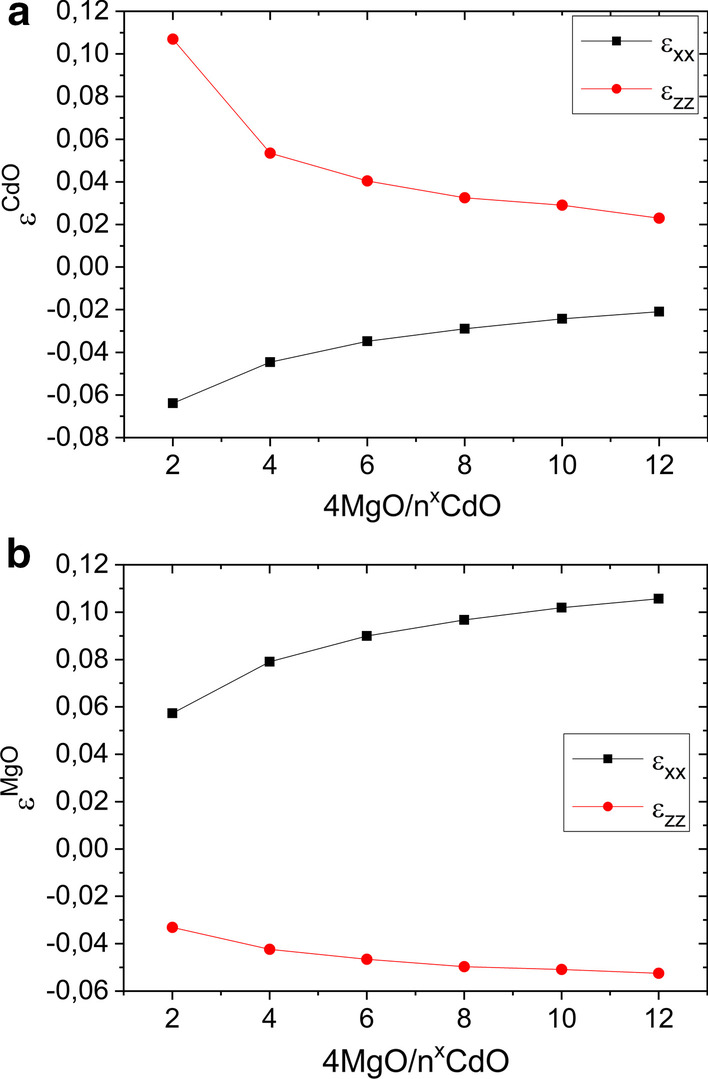
Calculated strain conditions for 4ML of MgO and various numbers of CdO monolayers structures: (**a**) in-plane (epsilon xx) and out-of-plane (epsilon zz) strains in CdO; (**b**) in-plane and out-of-plane strains in MgO

It is obvious that the strain affects the calculated band structure, on Fig. 7 we plot strain conditions realized in our structures. From the plots it follows that CdO layer are compressed in growth planes by MgO layers, this causes the material to stretch in the growth direction (Fig. 7a). On the other hand, we expect in-plane the tensile strain and out-of-plane compressive strain of the MgO layer (Fig. 7b).

## Comparison of Experiment and Theory

**Fig. 8:**
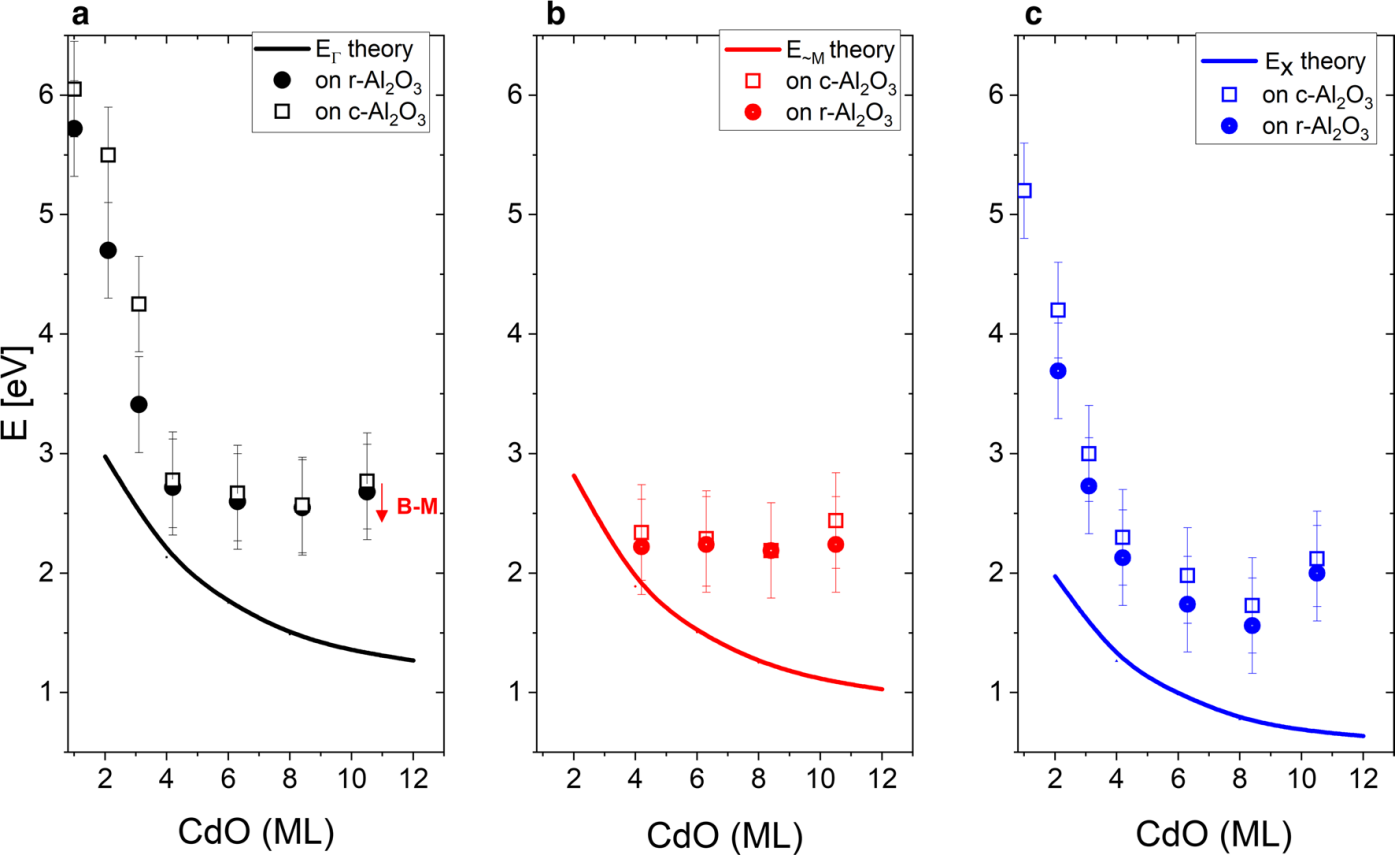
Comparison of the theoretical band gaps (solid lines) in Γ, *X* points and one maximum located close to *M* point and experimental data (symbols) obtained from transmittance data

In Fig. 8, the obtained band gap energies as a function of CdO layer thickness are compared with the results of our calculations. Our experimental points are marked as full for the 100 and open for the 111 orientation. Solid black, red and blue lines represent theoretically obtained values of direct and indirect band gaps in Γ, *X* and ~ *M* points. The experimental data are somewhat scattered, but reflect the theoretical trend. The experimental values of energy gaps are higher than those predicted theoretically. It should be noted that in the case of CdO-based layers, with a Cd-rich region, the electron concentration is usually high [57, 60]. It is well known that an increase in carrier density leads to the filling of states in the band, thus shifting the absorption onset to higher energies. This effect was independently discovered by Moss [61] and Burstein [62] in 1954 and is called the Burstein–Moss shift (BMS). Therefore, in CdO-based materials the band gap renormalization should be considered up to an electron density of about 9 × 10^18^ cm^−3^. We expect that the BMS will be higher for SL structures with a larger thickness of CdO layers. Likewise, the stress in SL layers can influence the measured band gap energies; as we know, in the case of thicker MgO and CdO sublayers the structure may be partially relaxed, whereas the calculations were made for fully strained SLs, i.e. single lattice constants were used for the whole structure, and we assumed that there were no dislocations or defects at the interfaces between the two sublattice materials. The Fermi energy was common for the whole structure and was in the middle of the energy gap, and so the free carrier concentration was set to zero. Calculated values of *B*-*M* shift in pure CdO for an electron concentration level of 2 × 10^20^ cm^−3^ are around 300 meV, and therefore for Cd-rich structures we should subtract certain values (< 300 meV) from the measured energy band gap.

**Fig. 9:**
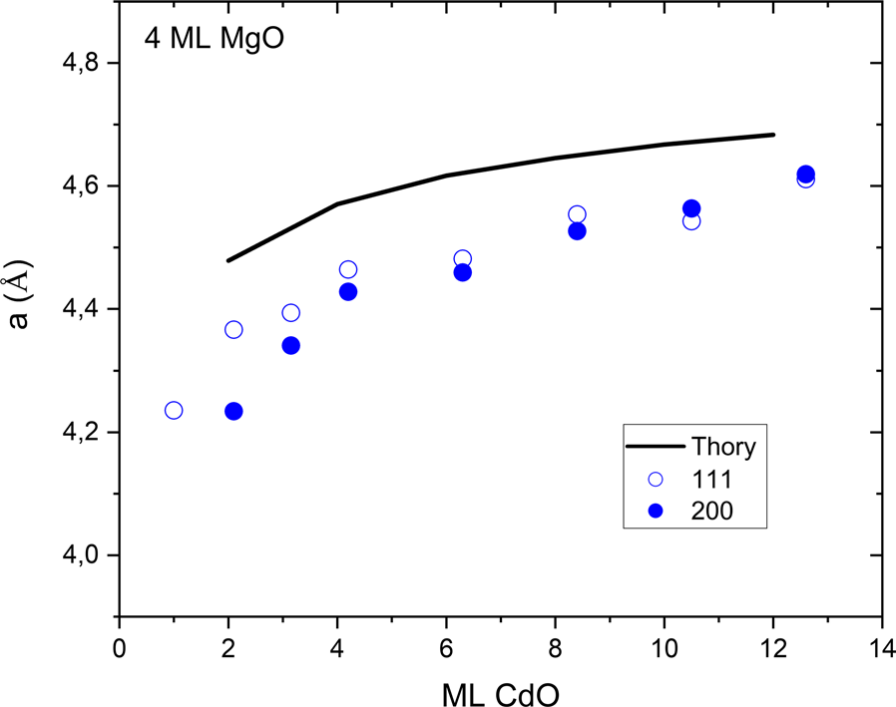
Comparison of the theoretical lattice constant (solid line) and experimental data (symbols: open for samples grown on 111 direction, full for samples grown on 001 direction) for series of SLs with different thickness of CdO sublayers

In the case of X-ray diffraction we also subtracted average lattice constants for measured SLs. The measured lattice constants increase with CdO sublayer thickness. The data obtained are compared with the theoretical calculations in Fig. 9. The experimental values are seen to be smaller than the calculated values, but the experimental data reproduce the theoretical trend.

## Conclusions

In conclusion, {CdO/MgO} quasi-alloys were synthesized by the MBE method in two crystallographic orientations. Their energy band gap and lattice constant properties were studied experimentally and calculated theoretically. The energy band gap of {CdO/MgO} quasi-alloys can be continuously modulated in a wide range from 2.6 to 6 eV by changing the thickness of the CdO sublattices. Correspondingly, the measured average lattice constants for {CdO/MgO} varied from 4.23 to 4.61 Å as the MgO thickness was kept constant and the CdO thickness was increased from 1 to 12 ML. The obtained values of the lattice constant are in good agreement with theoretical calculations, but are somewhat smaller than the calculated values, whereas the measured energy gaps are higher than those calculated ab initio for fully strained structures. The results show that the energy band gap of CdO can be tuned to higher values by using {CdO/MgO} quasi-alloys, and it is possible to engineer the energy gap over a wide range. This work has shown that {CdO/MgO} heterostructures can be useful in developing new optoelectronic devices, such as detectors for the visible, UV A, UV B and UV C regions.

## Data Availability

Not applicable.
